# Fumarate modulates the immune/inflammatory response and rescues nerve cells and neurological function after stroke in rats

**DOI:** 10.1186/s12974-016-0733-1

**Published:** 2016-10-13

**Authors:** Ruihe Lin, Jingli Cai, Eric W. Kostuk, Robert Rosenwasser, Lorraine Iacovitti

**Affiliations:** 1 The Joseph and Marie Field Cerebrovascular Research Laboratory at Jefferson, Vickie & Jack Farber Institute for Neurosciences, Department of Neuroscience, Sidney Kimmel Medical College, Thomas Jefferson University, 900 Walnut Street, Philadelphia, PA 19107 USA; 2 The Joseph and Marie Field Cerebrovascular Research Laboratory at Jefferson, Vickie & Jack Farber Institute for Neuroscience, Department of Neurological Surgery, Sidney Kimmel Medical College, Thomas Jefferson University, Philadelphia, PA 19107 USA

**Keywords:** Stroke, Inflammation, Dimethyl fumarate

## Abstract

**Background:**

Dimethyl fumarate (DMF), working via its metabolite monomethylfumarate (MMF), acts as a potent antioxidant and immunomodulator in animal models of neurologic disease and in patients with multiple sclerosis. These properties and their translational potential led us to investigate whether DMF/MMF could also protect at-risk and/or dying neurons in models of ischemic stroke in vitro and in vivo. Although the antioxidant effects have been partially addressed, the benefits of DMF immunomodulation after ischemic stroke still need to be explored.

**Methods:**

In vitro neuronal culture with oxygen-glucose deprivation and rats with middle cerebral artery occlusion were subjected to DMF/MMF treatment. Live/dead cell counting and LDH assay, as well as behavioral deficits, plasma cytokine assay, western blots, real-time PCR (Q-PCR) and immunofluorescence staining, were used to evaluate the mechanisms and neurological outcomes.

**Results:**

We found that MMF significantly rescued cortical neurons from oxygen-glucose deprivation (OGD) in culture and suppressed pro-inflammatory cytokines produced by primary mixed neuron/glia cultures subjected to OGD. In rats, DMF treatment significantly decreased infarction volume by nearly 40 % and significantly improved neurobehavioral deficits after middle cerebral artery occlusion (MCAO). In the acute early phase (72 h after MCAO), DMF induced the expression of transcription factor Nrf2 and its downstream mediator HO-1, important for the protection of infarcted cells against oxidative stress. In addition to its antioxidant role, DMF also acted as a potent immunomodulator, reducing the infiltration of neutrophils and T cells and the number of activated microglia/macrophages in the infarct region by more than 50 % by 7–14 days after MCAO. Concomitantly, the levels of potentially harmful pro-inflammatory cytokines were greatly reduced in the plasma and brain and in OGD neuron/glia cultures.

**Conclusions:**

We conclude that DMF is neuroprotective in experimental stroke because of its potent immunomodulatory and antioxidant effects and thus may be useful as a novel therapeutic agent to treat stroke in patients.

**Electronic supplementary material:**

The online version of this article (doi:10.1186/s12974-016-0733-1) contains supplementary material, which is available to authorized users.

## Background

In ischemic stroke, which is the leading cause of disability and the second leading cause of death worldwide [1], neuronal destruction is caused both by oxygen deprivation [2, 3] and by persistent activation of the host immune system [4–32]. After vessel occlusion, the loss of neurons occurs chiefly from the lack of oxygen supply and concomitant oxidative stress in the ischemic core. However, neuronal death in the surrounding penumbra area subsequently develops in large part from inflammation due to the infiltration of immune cells and the release of destructive cytokines. Thus, a novel regimen which enhances both cellular resistance to oxidative stress and modulation of the immune response could provide greater neuroprotection after ischemic stroke.

Intriguingly, the methyl ester of fumaric acid, dimethyl fumarate (DMF), working via its metabolite monomethylfumarate (MMF), acts as both a potent immunomodulator and antioxidant in laboratory models of disease [33–41] and in patients with neurological disease like multiple sclerosis (MS) [42, 43]. Moreover, several recent reports showed that DMF can also reduce brain edema and improve blood-brain barrier (BBB) integrity and improve neurological outcomes in a short-term rat model of hemorrhagic stroke [40, 44] and ischemic stroke [45, 46]. In all of these cases, DMF/MMF is thought to act via activation of the antioxidant transcription factor (erythroid-derived 2)-like 2 (Nrf2) which up-regulates proteins like heme oxygenase-1 (HO-1) [33, 36–38, 40, 41, 44, 47], thereby protecting cells against damage triggered by oxidant insult. Additionally, DMF is a potent modulator of inflammatory cytokines which are now known as important in stroke [11, 15, 20–22, 26, 27, 48, 49], in particular Th1-type pro-inflammatory cytokines that can lead to tissue damage [37, 38, 40, 50, 51].

Because of its acute antioxidant and prolonged immunomodulatory mechanisms of action and its translational potential in humans, we wondered whether DMF/MMF could protect both dying neurons in the ischemic core as well as neurons at-risk of dying at later times after stroke. We show here that MMF is neuroprotective to cortical neurons after oxygen-glucose deprivation (OGD) in a model of stroke in culture. In addition, in a rat model of experimental stroke, DMF significantly decreased infarction volume and improved neurobehavioral deficits 14 days after middle cerebral artery occlusion (MCAO), concomitant with the induction of Nrf2 and HO-1 and the reduction in immune cell infiltration and harmful inflammatory cytokines in the plasma and brain.

## Methods

### Animals

All procedures in this study were carried out in accordance with the recommendations in the Guide for the Care and Use of Laboratory Animals of the National Institutes of Health. The protocol was approved by the IACUC Committee of Thomas Jefferson University.

### Antibodies and reagents

Dimethyl fumarate (DMF) and mono-methyl fumarate (MMF) were purchased from Sigma-Aldrich. Poly-d-lysine, DMSO and Glutamax were purchased from Sigma. HBSS, DMEM, fetal calf serum, Neurobasal medium, B-27 supplement were purchased from Invitrogen. LIVE/DEAD viability/cytotoxicity kit was purchased from Molecular Probes. The cytotoxicity detection kit plus (lactate dehydrogenase (LDH)) was purchased from Roche. The following primary antibodies were used in these experiments: rabbit anti-Nrf2 (1:200, Santa Cruz Biotechnology), rabbit anti-HO-1 (1:4000, Enzo Life Sciences), rabbit anti-β-tubulin (1:1000, Cell Signaling Technology), mouse anti-rat CD-3 (1:25, BD Biosciences), rabbit anti-myeloperoxidase (MPO) (1:300, DAKO), mouse anti-CD-68 (1:100, Abcam), and rabbit anti-iNOS (1:80, Abcam).

### Primary cortical cultures

Primary cultures of rat forebrain neurons were used for LIVE/DEAD assay and LDH releasing assay. The cultures were prepared from embryos of Sprague-Dawley rats at day 15 of gestation. The cells were dissociated in dissociation buffer with Papain and DNase I. The cell suspension was passed through a cell strainer and then centrifuged. The pellet was re-suspended in high-glucose DMEM containing 10 % fetal calf serum and plated onto glass coverslips pre-coated with poly-d-lysine in 12-well plates at a final concentration of 2.5 × 10^5^ cells/ml. At 24 h after seeding, the medium was changed to Neurobasal medium supplemented with B-27 and Glutamax. The cells were cultured at 37 °C in a humidified atmosphere of 95 % air and 5 % CO_2_. Half of the culture medium was replaced with fresh Neurobasal/B-27 medium twice a week. The cells were tested after 5–7 days in vitro when most exhibited a neuronal morphology.

Primary mixed neuron/glia cultures of rat cortex were used for real-time PCR (Q-PCR) analysis. The cultures were prepared from embryos of Sprague-Dawley rats at day E15-E17.5 of gestation as described previously [2, 52]. The cells were plated onto glass coverslips and cultured in DMEM/F12 supplemented with 5 % FBS, 5 % HS and 1 % P/S (*v*/*v*) after dissociation (Papain and DNase I) and re-suspension as above. The cells were cultured at 37 °C in a humidified atmosphere of 95 % air and 5 % CO_2_. The culture medium was replaced twice per week, and the cells were analyzed by Q-PCR after 7–10 days in vitro.

### Oxygen-glucose deprivation

MMF was dissolved in DMSO as a 50-mM stock solution and stored in aliquots at −20 °C. In pretreated groups, the stock solution of MMF was serially diluted in the respective culture medium and cultures were pretreated for 12 h before OGD. Cell cultures were then washed twice with HBSS and OGD media (glucose and phenol red-free DMEM was deoxygenated by gassing with 95 % nitrogen and 5 % CO_2_ for 15 min) containing either 25, 50, or 100 μM MMF. Untreated control cells received DMSO vehicle only. Cultures were then put in a sealed chamber (Billups-Rothenberg, Inc., Del Mar, CA, USA), flushed with 95 % nitrogen and 5 % CO_2_ for 6 min at a flow rate of 20 L/min and incubated for 1 h (primary cortical cultures for LIVE/DEAD assay) and 2 h (mixed neuron/glia cultures for Q-PCR) at 37 °C. The time points were selected based on our empirical data, the literature [2], and MCAO procedure. OGD was terminated by removing the cell culture plates from the chamber, washing, and replacing the glucose-free medium with the respective culture medium with MMF at corresponding concentrations. The cells were then returned to a regular 5 % CO_2_ incubator. In posttreatment groups, the stock solution of MMF was diluted in the respective culture media and cultures were incubated with MMF after OGD. At 24 and 48 h after OGD, the media from neuronal culture was collected for LDH assay and cells were processed using the LIVE/DEAD assay to determine cellular viability. At 24 h after OGD, RNA from mixed cortical culture was extracted for real-time PCR analysis.

### LIVE/DEAD assay

Neuronal viability was quantified using a LIVE/DEAD viability/cytotoxicity kit (Molecular Probes). According to the manufacturer’s protocol, coverslips were stained with calcein AM and ethidium homodimer-1, which labeled live cells and dead cells, respectively. Coverslips were then quickly examined under a fluorescence microscope (Olympus IX2-UCB), and pictures were taken. Neuronal death was determined by counting from six random fields per coverslip then averaged and expressed as percentage of cell death (i.e., dead cells/total cells × 100 %). All assays were repeated in triplicate in three independent experiments.

### LDH releasing assay

Cell injury was quantitatively assessed by measuring lactate dehydrogenase (LDH) in the media, as previously reported [53]. Briefly, cell-free culture medium was collected, and the amount of LDH released into the media was measured by using the Cytotoxicity Detection Kit Plus (Roche) at 24 h after OGD according to the manufacturer’s protocol. Results were read on a Bio-Rad plate reader. Percent cell injury was determined as experimental LDH release/total LDH release after lysis buffer-induced death × 100 after correcting for baseline absorbance. All assays were repeated in triplicate in three independent experiments.

### Focal ischemic stroke: MCAO

Adult male Sprague-Dawley rats weighing 275–300 g were anesthetized, and MCAO was performed as previously reported [54, 55]. For details, adult male Sprague-Dawley rats weighing 275–300 g were anesthetized using SQ ketamine hydrochloride, xylazine, and acepromazine maleate (60, 10, and 5 mg/kg, respectively). Body temperature was monitored with a rectal temperature probe and maintained with a heating pad and/or a small fan to within 0.5 °C. Briefly, the right common carotid (CCA) and external and internal carotid arteries (ECA, ICA) were exposed, and the right ECA was ligated. The right CCA was ligated at the proximal end, and the right ICA blood flow was then blocked by clamping using a micro-clip at its origin. A silicone rubber-coated nylon filament (Doccol) was then inserted into the lumen of the CCA through a small opening. The clamp on the right ICA was then removed, and the nylon filament was carefully advanced into the ICA until it obstructed the middle cerebral artery (MCA). Two hours later, the nylon filament was removed and CCA was ligated to stop bleeding and allow reperfusion of the brain. Mortality rates and poststroke body weight were recorded for all experimental groups. In order to examine the efficiency and tolerance of the DMF in the stroke models and evaluate its effects without bias, no animal was excluded from endpoint analysis unless due to death (<2 %).

### Animal treatment protocol

Adult male Sprague-Dawley rats weighing 275–300 g were used in these experiments. For behavioral tests, rats were divided into four various control and DMF treatment groups. Treatments were administered by twice daily oral gavage. Group 1 served as normal (Nor) control without MCAO procedure. Group 2 rats were subjected to MCAO and served as control (Veh) for MCAO DMF treatment. Group 3 (dosage = 25 mg/kg) and group 4 (dosage = 50 mg/kg) served as DMF treatment groups with oral gavage beginning 2–3 h after MCAO surgery. In groups 3 and 4, rats received either 25 or 50 mg/kg of DMF in 0.08 % methocel solution (DMF-treated group) and in group 2, the same amount of methocel solution was used as control. At postoperative 24 h, 72–84 h, day 7, and day 14, four rats from each group were first evaluated by behavioral tests and then deeply anesthetized. The plasma and brains were collected from some animals in groups 1, 2, and 4 rats for RT-qPCR and Western blot analysis while other animals were transcardially perfused with cold 4 % paraformaldehyde for immunocytochemical analysis.

### Behavioral tests

To evaluate neurological function, all rats were subjected to a battery of tests at postoperative 24 h, 72–84 h, day 7, and day 14 after MCAO (groups 1–4 above). Motor and sensory deficits were evaluated using a modified neurological severity score (mNSS). Using this scale, one point was given for the inability to perform a test. Consequently, the higher the score, the more severe is the deficit (maximum score = 18). Behavior was assessed at regular intervals by an observer blinded to treatment status.

### Evaluation of infarction volume

Brains were harvested 72 h after MCAO from control and DMF-treated groups (dosage = 25 or 50 mg/kg). Each brain was sliced into five coronal sections (thickness = 2 mm) and then processed for staining with 2 % 2,3,5-triphenyltetrazolium chloride (TTC; Sigma, St. Louis, MO, USA) in saline as described previously. After incubation in 2 % TTC for 20 min at 37 °C, the brain slices were then fixed in 10 % formalin for 24 h at room temperature. Infarct volume was measured and calculated by using digital imaging and NIH Image program (ImageJ software). To control for edema, infarct volume was determined by subtraction of the ipsilateral viable (TTC+) regional volume from the corresponding contralateral viable (TTC+) counterpart. This value was then divided by the contralateral value and then multiplied by 100 and expressed as a percent of the contralateral viable volume.

### Multiplex cytokine assays

Plasma was obtained from anticoagulated cardiac blood samples from group 1, 2, and 4 rats. The assay was performed using the instructions provided by DartLab (Immunoassay and Flow Cytometry Shared Resource at the Geisel School of Medicine at Dartmouth) as follows. Briefly, cytokines were measured using Millipore rat cytokine multiplex kits (EMD Millipore Corporation, Billerica, MA). Calibration curves from recombinant cytokine standards were prepared with threefold dilution steps in the same matrix as the samples. High and low spikes were included to determine cytokine recovery. Standards and spikes were measured in triplicate, samples were measured in duplicate, and blank values were subtracted from all readings. All assays were carried out directly in a 96-well filtration plate (Millipore, Billerica, MA) at room temperature and protected from light. Briefly, wells were pre-wet with 100 μl PBS containing 1 % BSA, and then beads together with a standard, sample, spikes, or blank were added in a final volume of 100 μl, and incubated at room temperature for 30 min with continuous shaking. Beads were then washed three times with 100 μl PBS containing 1 % BSA and 0.05 % Tween 20. A cocktail of biotinylated antibodies (50 μl/well) was added to beads for a further 30-min incubation with continuous shaking. Beads were washed three times, and then streptavidin-PE was added for 10 min. Beads were again washed three times and re-suspended in 125 μl of PBS containing 1 % BSA and 0.05 % Tween 20. The fluorescence intensity of the beads was measured using the Bio-Plex array reader. Bio-Plex Manager software with five-parametric-curve fitting was used for data analysis.

### Western blot analysis

Rats from groups 1, 2 and 4 were briefly perfused transcardially with 0.9 % saline, and the right (ipsilateral) cerebral cortex and striatum (from Bregma 2.5 to −7.5 mm) were dissected and homogenized in lysis buffer containing protease inhibitors. After centrifugation (17,000 g for 30 min), supernatants were collected and protein concentration was determined. Equal amounts of protein (20 μg) were separated by SDS/PAGE (NuPAGE precast polyacrylamide gel) and transferred onto nitrocellulose membrane (Millipore). Membranes were blocked for 1 h in Tris-buffered saline (TBST), with 0.1 % Tween-20 and 5 % non-fat dry milk, followed by an overnight incubation with primary antibody diluted in the same buffer. Blots were incubated with the appropriate primary antibodies: anti-Nrf2 (1:200), anti-HO-1 (1:4000), or anti-β-III tubulin (1:1000). After washing with TBST, the membrane was incubated with peroxidase-conjugated secondary antibody for 1 h and then washed and developed using the ECL chemiluminescent detection system. Densitometric analyses were performed using the NIH Image program (ImageJ software), and the ratio between the protein and the corresponding loading control was calculated.

### RNA isolation and cDNA synthesis

In OGD experiments performed in culture, total RNA was isolated directly from primary cortical mixed neuron/glia cultures with TRIzol (invitrogen). Rats from groups 1, 2, and 4 were briefly perfused transcardially with 0.9 % saline, and total RNA was isolated directly from the right (ipsilateral) hemisphere including cerebral cortex and striatum (from Bregma 2.5 to −7.5 mm) with TRIzol. Complementary DNA (cDNA) was synthesized by using 1 μg total RNA in a 20 μl reaction with Superscript III (invitrogen) and oligo (dT) 18 (invitrogen). One microliter of RNase H (invitrogen) was added to each reaction tube, and the tubes were incubated for 20 min at 37 °C before proceeding to PCR.

### Real-time PCR analysis

Real-time PCR was carried out by 7500 Real-Time PCR System using SYBR green PCR master mix (both from Applied Biosystems). CYPA was used as an internal control. All PCR products were checked by running an agarose gel for the first time and by doing dissociation assay every time to exclude the possibility of multiple products. PCR analyses were conducted in triplicate for each sample. Primers for real-time PCR were designed by using the Primer-BLAST and are listed as Table 1.

**Table 1:** Primer sequences

Gene	Sequence
IL-12A(p35)	forward 5′-TGTCAATCACGCTACCTCCTC-3′
	reverse 5′-AAGACACTTGGCAGGTCCAG-3′
IL-12B(p40)	forward 5′-TGGGAGTACCCTGACTCCTG-3′
	reverse 5′-AGGAACGCACCTTTCTGGTT-3′
IP-10 (CXCL10)	forward 5′-CCGCATGTTGAGATCATTGCC-3′
	reverse 5′-TCTTTGGCTCACCGCTTTCA-3′
IFN-γ	forward 5′-GCAAAAGGACGGTAACACGA-3′
	reverse 5′-TTGCTGATGGCCTGGTTGTC-3′
IL-23A(p19)	forward 5′-GACTAAAAGTGACGTGCCCC-3′
	reverse 5′-AAACAGAACTGGCTGTTGTCC-3′
IL-18	forward 5′-ACCGCAGTAATACGGAGCAT-3′
	reverse 5′-TCTGGGATTCGTTGGCTGTT-3′
IL-1β	forward 5′-GGCTTCCTTGTGCAAGTGTC-3′
	reverse 5′-AGTCAAGGGCTTGGAAGCAA-3′
TNF-α	forward 5′-ATGGGCTCCCTCTCATCAGT-3′
	reverse 5′-GCTTGGTGGTTTGCTACGAC-3′
MIP-2	forward 5′-CTGAACAAAGGCAAGGCTAACT-3′
	reverse 5′-TTGATTCTGCCCGTTGAGGT-3′
EOTAXIN	forward 5′-TTCTATTCCTGCTGCTCACGG-3′
	reverse 5′-GTTGGGATGGAACCTGGGTG-3′
RANTES (CCL5)	forward 5′-GTGCCCACGTGAAGGAGTAT-3′
	reverse 5′-TCGAGTGACAAAGACGACTGC-3′
IL-17	forward 5′-ATCCATGTGCCTGATGCTGTT-3′
	reverse 5′-AAGTTATTGGCCTCGGCGTT-3′
IL-5	forward 5′-TGTTGACGAGCAATGAGACGA-3′
	reverse 5′-CCCCCTCGGACAGTTTGATT-3′
IL-10	forward 5′-TGCGACGCTGTCATCGATTT-3′
	reverse 5′-TGGCCTTGTAGACACCTTTGT-3′
GM-CSF	forward 5′-ATACAAGCAGGGTCTACGGG-3′
	reverse 5′-GTCAGTTTCCGGGGTTGGA-3′
VEGF-A7	forward 5′-CACCATGCCAAGTGGTGAAG-3′
	reverse 5′-AGATGTCCACCAGGGTCTCA-3′
MCP-1 (CCL2)	forward 5′-TGTCTCAGCCAGATGCAGTTAAT-3′
	reverse 5′-TCCAGCCGACTCATTGGGAT-3′
CYPA	forward 5′-TATCTGCACTGCCAAGACTGAGTG-3′
	reverse 5′-CTTCTTGCTGGTCTTGCCATTCC-3′
GAPDH	forward 5′-CAACTCCCTCAAGATTGTCAGCAA-3′
	reverse 5′-GGCATGGACTGTGGTCATGA-3′

### Immunostaining/cell quantification

Animals were perfused with cold (4 °C) paraformaldehyde (4 %). Brains were postfixed in 4 % paraformaldehyde at 4 °C for 24–36 h, immersed in 30 % sucrose solution at 4 °C, and then embedded in OCT (Tissue-Tek, Sakura, Japan) before cutting with a cryostat (Microm HM505E). Coronal sections were cut at 20 μm on a cryostat and collected onto slides. After antigen retrieval, sections were incubated with primary antibodies in blocking buffer containing 0.1 % Triton × 100 and 5 % normal donkey serum (NDS) in 0.01 M phosphate-buffered saline (pH 7.4). Sections were incubated with primary antibodies (anti-MPO, anti-CD3, anti-CD68, and anti-iNOS) for 48 h at 4 °C, washed, and incubated with secondary antibodies for 2 h at room temperature in blocking buffer. The nuclear dye DAPI was added after the secondary antibody incubation. Sections were then cover-slipped and examined, and images were acquired using an Olympus IX81 Image Analysis System or laser confocal microscopy (Olympus Fluoview). Sections from similar ischemic brain regions from groups 2 and 4 (*n* = 6, 5 sections from each brain) were used for cell quantification. Images of three microscopic fields in the penumbra region of each section were randomly acquired under ×100 magnification and immunopositive cells were counted and expressed as cell number/field.

### Statistical analysis

All data are presented as the mean ± SEM. The statistical significance of the mean was calculated by the Mann-Whitney *U* test or Student’s *t* test. A value of *p* < 0.05 was considered significant.

## Results

### MMF rescues forebrain neurons from OGD-induced cell death in culture

**Fig. 1:**
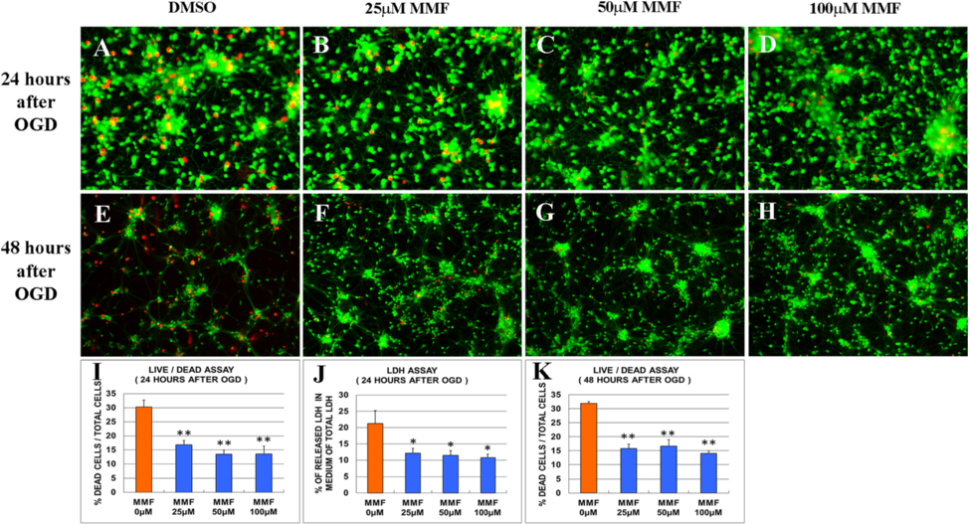
MMF rescues forebrain neurons from OGD-induced cell death in culture. Cultures of E15 rat forebrain were treated with MMF (25, 50, or 100 μM) beginning 12 h prior to OGD deprivation and throughout the remaining culture period. Cultures treated with MMF contained significantly fewer dead/dying cells at 24 h (**a**–**d**, **i**) and 48 h (**e**–**h**) than untreated controls as assessed by LIVE/DEAD assay (**a**–**i**, **k**) or assay of LDH released into the media by dead cells (**j**). *p < 0.05; **p < 0.01, Student’s *t* test

In a model simulating ischemia in culture, forebrain neurons were isolated from E15 rat embryos and maintained in culture for 7 days before depriving them of oxygen and glucose (OGD) for 1 h. To test the neuroprotective effects of MMF, cultures were treated varying concentrations of MMF (25–100 μM), beginning 12 h prior to OGD and continuing throughout the duration of the experiment, and compared to DMSO controls. In posttreatment groups, cultures were incubated with MMF (25 μM) or DMSO after 1 h of OGD. Neuronal survival was then assessed by the LIVE/DEAD assay or by assessing LDH activity in the culture media 24–48 h later. In posttreatment groups, we found fewer dead (red) cells at 24 h in cultures treated with 25 μM MMF (28.5 % in controls vs 21.1 % in MMF treated) (Additional file 1: Figure S1A-C), but there was no significant difference between the two groups when assessed by LDH assay (Additional file 1: Figure S1D). In pretreatment groups, we found significantly fewer dead (red) cells at 24 h in cultures treated with 25, 50, or 100 μM MMF (27.4 % in controls vs 13.6 % in 100 μM MMF treated) (Fig. 1a–d, i). Similarly, the amount of LDH released into the media by presumptive dead or dying cells was significantly lower in MMF-treated cultures (21.2 % in controls vs 10.8 % in 100 μM MMF treated) (Fig. 1j). Importantly, with continual treatment, MMF-enhanced neuronal rescue was sustained even at later times (48 h post-OGD) (29.5 % in controls vs 14.0 % in 100 μM MMF treated) (Fig. 1e–h, k). Thus, in culture, pretreatment of cells with MMF before OGD was significantly more effective at rescuing dying cells than treatment after OGD.

### DMF reduces infarction size and reduces neurobehavioral deficits after MCAO

**Fig. 2:**
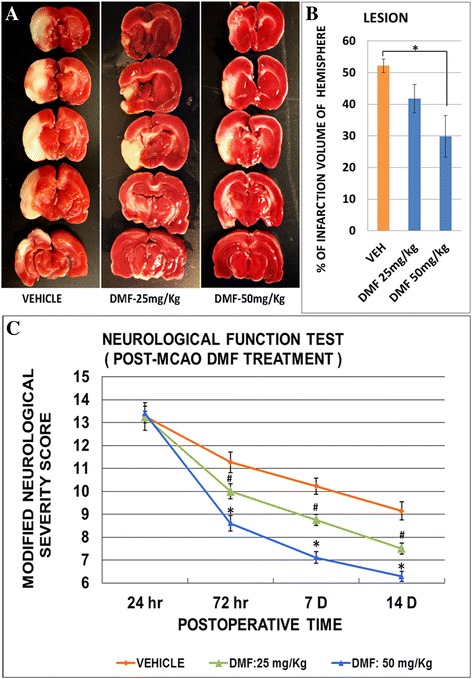
Infarction size and behavioral assessment after MCAO and DMF treatment. Control (vehicle treated, *n* = 8) and DMF (25 or 50 mg/kg, *n* = 8 of each group)-treated rat brains were sectioned and stained for TTC 72 h after MCAO (**a**). Infarction volume was then calculated as described in the “Methods” section using ImageJ and expressed as a percentage of total hemisphere (**b**). mNSS was assessed at 24 h, 72–84 h, 7 days, or 14 days after MCAO during which rats received either vehicle or 25 or 50 mg/kg DMF by oral gavage beginning 2–3 h after MCAO (*n* = 8 of each group). A significant and long-lasting decline in neurobehavioral deficits was seen 72 h, 7 days, and 14 days after the initiation of DMF treatment as compared to controls (**c**). *, ^#^ *p* < 0.05, Mann-Whitney *U* test

To test DMF effects in vivo, rats were administered saline or DMF (25 or 50 mg/kg) twice daily via oral gavage, beginning 2–3 h after MCAO and continuing until the completion of the experiment. The average weight loss was 30–35 g in the control group and 40–50 g in the DMF-treated group at the 14-day time point. Animals were also subjected to neurobehavioral function tests before and 24 h, 72–84 h, and 7 and 14 days after MCAO using a modified neurological severity score (mNSS). Upon sacrifice, the brain was stained with TTC, and infarction volumes were calculated 3 days after MCAO. We found that DMF significantly reduced the size of infarction in TTC-stained sections from 52.2 % in untreated controls at 72 h after MCAO to 41.8 % (25 mg/kg) and 29.9 % (50 mg/kg) in DMF-treated rats (Fig. 2a, b). Correlated with smaller infarction volumes, we observed a significant improvement in neurobehavioral scores (mNSS) in animals administered DMF compared to controls (vehicle treatment) (Fig. 2c). Interestingly, although mNSS scores were nearly identical at the start, by 72 h after the initiation of either 25 or 50 mg/kg DMF treatment, scores declined in a dose- and time-dependent manner. Although the greatest behavioral improvement was seen in the first 72 h after DMF, mNSS scores continued to gradually decline with longer treatment times (up to 14 days, the latest time point examined). These results demonstrate that 25 mg/kg DMF is less effective than 50 mg/kg in the MCAO model when neurological behavior test and infarct size (i.e., TTC staining) are assessed. Based on these findings, we used 50 mg/kg DMF in our subsequent vivo studies.

### DMF induces Nrf-2 and its downstream effector HO-1 in the MCAO rat

**Fig. 3:**
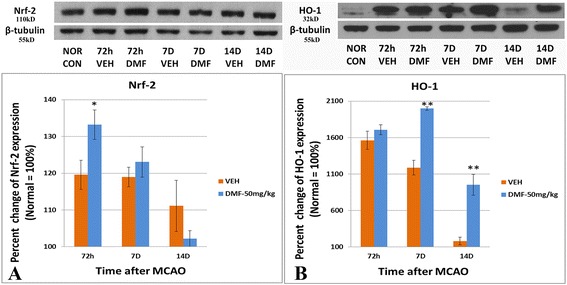
Western analysis of Nrf-2 and HO-1 after MCAO and DMF treatment. Protein levels of Nrf-2 (**a**) and HO-1 (**b**) were measured by Western analysis after vehicle (veh) or 50 mg/kg DMF treatment for 72 h, 7 days, or 14 days after MCAO (*n* = 4 of each group). All values were expressed as percent change HO-1 expression over normal rat brain (100 %). Significant differences are shown for MCAO/DMF as compared to MCAO/veh. *p < 0.05; **p < 0.01, Mann-Whitney *U* test

As DMF (via MMF) is believed to activate Nrf-2, which regulates a host of downstream effector molecules important in limiting oxidant damage, including HO-1 [33, 36–38, 40, 41, 44, 47], we next examined Nrf-2 and HO-1 protein levels in control (vehicle) and DMF-treated MCAO rats and compared levels in the hemisected brain on the side of the injury to normal uninjured hemisphere. We found MCAO itself significantly increased Nrf-2 and HO-1 levels. Importantly, however, DMF treatment markedly increased Nrf-2 levels at 72 h compared to vehicle-treated controls and normal uninjured brain (Fig. 3a). Although HO-1 levels also increased after DMF treatment, unlike Nfr-2, the rise was not detected until 7 days after the treatment begun (Fig. 3b). While HO-1 remained elevated compared to MCAO-vehicle-treated controls at 14 days, levels were decreased from their peak at 7 days (Fig. 3b).

### DMF modulates in vitro and in vivo immune factors

In addition to acting as an antioxidant, DMF is also known to be a potent immunomodulator in a variety of diseases [33–43]. Therefore, we examined immune/inflammatory cytokines and growth factors in primary mixed neuron/glia cultures and in brain tissue and plasma in MCAO rats after MMF or DMF treatment.

**Fig. 4:**
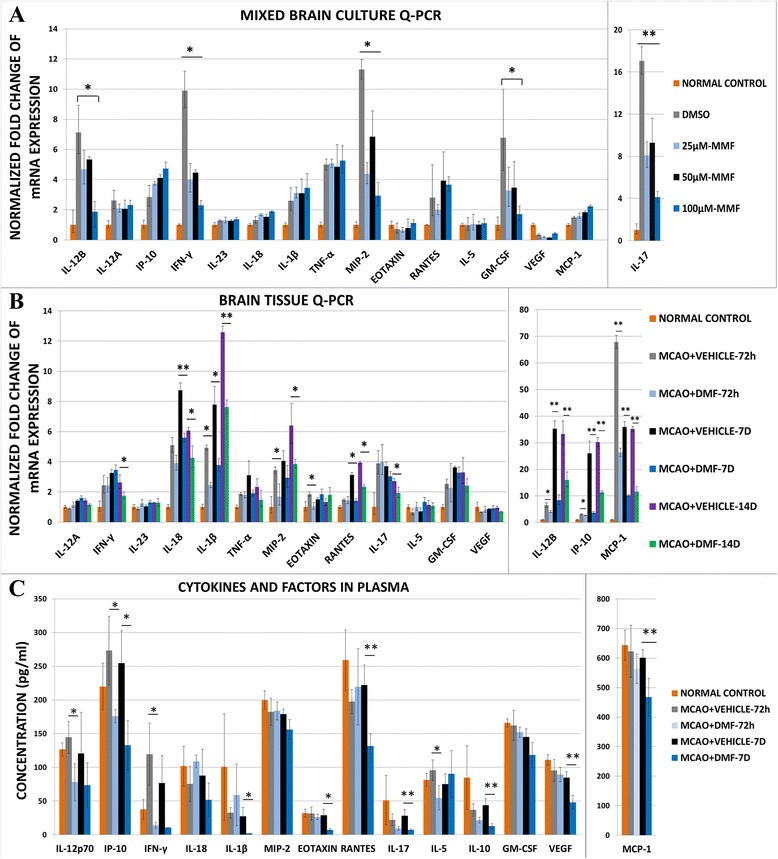
Cytokines and growth factors in mixed neuron/glia culture after OGD, brain and plasma after MCAO with or without MMF/DMF pretreatment. Cultures were processed for RT-qPCR and analyzed for cytokine mRNA levels 24 h after OGD (**a**). The side of the brain ipsilateral to MCAO was isolated 72 h, 7 days, and 14 days after surgery for RT-qPCR and analyzed for cytokine and growth factor mRNA levels (**b**, *n* = 4 of each group). In animals treated as in **b**, blood levels of factors were measured by multiplex array (see the “Methods” section) in normal uninjured brain or in MCAO rats after 72 h or 7 days of vehicle or 50 mg/kg DMF treatment; protein levels were expressed as pg/ml (**c**, *n* = 3–4 per group). All values were compared to normal uninjured control brain. *p < 0.05; **p < 0.01, Mann-Whitney *U* test

We first examined immune cytokine levels in OGD mixed neuron/glia cultures after treatment with vehicle or MMF using RT-qPCR. IL-12B, IFN-γ, IL-17, GM-CSF, MIP-2, IL-1β, and TNF-α were significantly induced after OGD. In contrast, after incubation with MMF, there was a significant reduction in IL-12B, IFN-γ, IL-17, GM-CSF, and MIP-2 (Fig. 4a).

We then examined messenger RNA (mRNA) expression levels of immune cytokines and growth factors in the hemisphere ipsilateral to MCAO as compared to the same hemisphere in normal brain in vehicle-treated and DMF-treated rats using Q-PCR. Nearly every factor tested was induced by MCAO alone. However, after 3 days of DMF treatment, a significant decline in IL-12p40, IP-10, IL-1β, MIP-2, eotaxin, and MCP-1 but surprisingly not IFN-γ was observed (Fig. 4b). The decrease in IL-12p40, IP-10, IL-1β, MIP-2, and MCP-1 was still observed 14 days after MCAO. Moreover, IL-18 mRNA level started to decrease 7 days after MCAO while IL-17 and IFN-γ were decreased by 14 days.

In addition to tissue cytokine levels, on the day of sacrifice, blood was collected (see the “Methods” section) and samples were sent for multiplex analysis of cytokines and growth factors. We found that experimental stroke significantly induced IFN-γ levels at 72 h post-MCAO and that the levels remained elevated even at 7 days post-MCAO (Fig. 4c). Importantly, following DMF treatment, IFN-γ was significantly decreased at 72 h when compared to the vehicle-treated MCAO brains or normal brain. In addition, DMF treatment also resulted in a decline in a number of other potentially deleterious inflammatory cytokines, including IL-12p70, IP10, and MCP-1, when compared to vehicle-treated MCAO brain levels. In some cases (IP-10, MCP-1), these early reductions were sustained with continued DMF treatment for 7 days (Fig. 4c). Interestingly, DMF also resulted in a reduction IL-1β, IL-17, eotaxin, RANTES, and VEGF levels in plasma at 7 days, even though these factors were unchanged at 72 h (Fig. 4c). Thus, DMF treatment begun within hours of MCAO and continued thereafter results in both early and late effects on plasma cytokines, causing a profound down-regulation of potentially deleterious inflammatory factors.

### DMF reduces immune cell infiltration and microglial activation in the infarct region

**Fig. 5:**
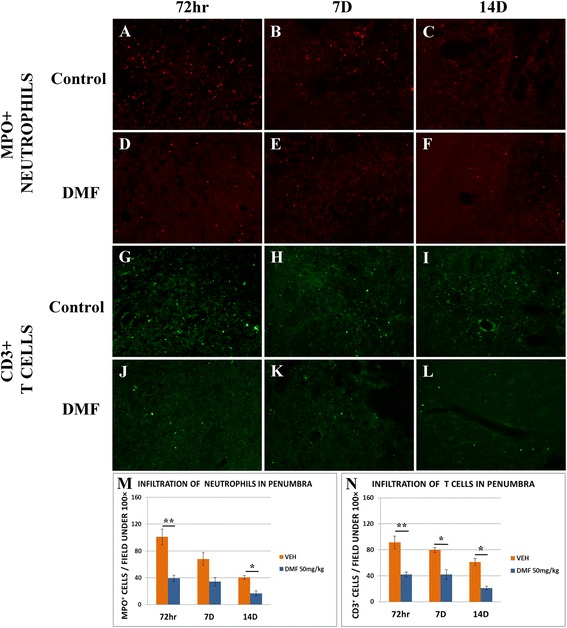
Analysis of immune cell infiltration into the infarct region 72 h, 7 days, and 14 days after MCAO (*n* = 6 of each group). Neutrophil cells in the infarct region were stained for MPO in control (**a**–**c**) and DMF treated (**d**–**f**) MCAO rats and quantified (M). T cells were stained for CD3 in control (**g**–**i**) and DMF treated (**j**–**l**) MCAO rats and quantified (N). *p < 0.05, **p < 0.01, Mann-Whitney *U* test

Supporting the notion of DMF-mediated immunomodulation in the brain, we found that the number of MPO+ neutrophils (Fig. 5a–f, m) and CD3+ T cells (Fig. 5g–l, n), which had infiltrated the penumbra region surrounding the infarct, was significantly lower in DMF-treated compared to vehicle-treated (control) MCAO rats.

**Fig. 6:**
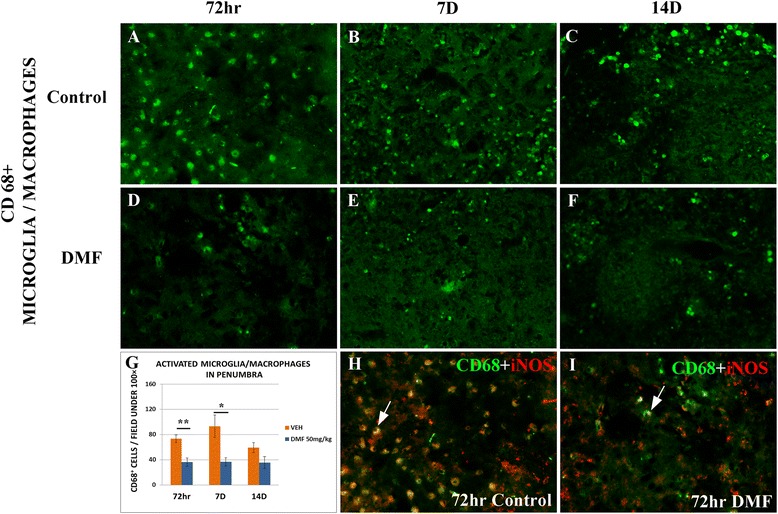
Analysis of activated microglia/macrophages in penumbra 72 h, 7 days, and 14 days after MCAO (*n* = 6 of each group). Activated microglia/macrophages were stained for CD68 in control (**a**–**c**) and DMF treated (**d**–**f**) MCAO rats, and cell number was quantified (**g**). iNOS staining was also used to demonstrate activated microglia/macrophages at 72 h (**h**, **i**, *arrows*). *p < 0.05, **p < 0.01, Mann-Whitney *U* test

Additionally, DMF treatment greatly reduced the number of activated CD68+ microglia/macrophages in the penumbra region after MCAO. Thus, in vehicle-treated MCAO rats, the number of CD68+ microglia/macrophages increased dramatically (Fig. 6a–c) compared to normal control rats where relatively few CD68+ cells were observed (data not shown). Importantly, DMF treatment greatly suppressed this inductive effect (Fig. 6d–f), decreasing more than 50 % of CD68+ cells/microscopic field (*p* ≤ 0.01) at 72 h (Fig. 6g). The vast majority of CD68+ cells also stained positive with iNOS at 72 h (Fig. 6h, i, arrows), another marker of activated microglia [18].

## Discussion

This study demonstrated for the first time that DMF/MMF has a profound combined antioxidant and anti-inflammatory effect, greatly increasing the short (72 h)- and long (7 and 14 days)term rescue of dying and “at-risk” brain neurons in models of ischemic stroke in vivo and in vitro. In cultures, MMF protected cortical neurons from damage and death due to transient oxygen-glucose deprivation. This was due in part to the down-regulation of potentially deleterious cytokines, consistent with the previous reports [40, 44–46]. In vivo, DMF treatment initiated within several hours of experimental stroke greatly diminished infarction volume and ameliorated behavioral deficits after MCAO. At the molecular level, DMF treatment induced the transcription factor Nrf-2 and its downstream effector HO-1 in the MCAO brain, both of which are known to be critically important for constraining oxidant damage acutely after stroke [56–59]. Also important and contributing further to the observed beneficial outcome were the long-term changes in the immune landscape of the brain after DMF treatment. Thus, DMF greatly reduced the infiltration of potentially damaging blood immune cells into the infarct, such as CD3+ T cells and MPO+ neutrophils, and decreased the levels of harmful inflammatory cytokines in the blood and brain. Furthermore, DMF greatly suppressed the activation of microglia in the penumbra and surrounding brain region.

DMF, which was recently approved by the FDA for the treatment of patients with relapsing-remitting MS [60], has been well characterized previously in animal models of MS [61] and several other neurological disorders [37, 39] including hemorrhagic stroke [40, 44] and models of ischemia [45, 46]. These studies have led to the widespread belief that the molecular basis for the drug’s efficacy is the transcription factor Nrf-2 [33, 36, 37, 44, 46, 47] and its regulation of key target genes involved in cellular antioxidant and defense mechanisms [62–64]. Unlike previous studies where analyses were confined to the acute phase (i.e., up to 72 h after stroke), in the present study, neurological behavior along with the brain and blood was analyzed both in the short and long term after DMF treatment.

We found major behavioral improvement in MCAO rats as early as 72 h after the administration of DMF. Importantly, during this early acute phase in MCAO rats, brain levels of Nrf-2 also increased but interestingly, changes in its crucial downstream mediator HO-1 did not peak until later (7 days after the initiation of treatment), possibly indicating different mechanisms for the early rescue by DMF of dying core cells and the later protection of at-risk penumbra cells.

In particular, the long-term neuroprotective effects of DMF raised the possibility that factors/pathways other than Nrf-2/HO-1 and mechanisms other than antioxidant systems might be involved. This contention was further supported by the findings of Zamvil and colleagues showing that Nrf2 does not mediate all activities of fumarates [65]. Since the prolonged harmful consequences of inflammation after stroke are well known [6, 11], we examined the immunomodulatory function of DMF in stroke. Indeed, we found that DMF treatment had profound effects on plasma and brain immune cytokines in rats after MCAO as well as in OGD mixed neuron/glia cultures. Of particular note was the dramatic down-regulation in levels of the pro-inflammatory cytokine IFN-γ in culture and in plasma.

In addition to IFN-γ, DMF also significantly reduced the levels of IL-12 in plasma and IL-12p40 in the brain, likely leading to a further down-regulation of IFN-γ and its downstream mediator IP-10. Likewise, in OGD mixed neuron/glia cultures, DMF decreased IL-12p40 levels. IL-12, which is produced by activated microglial factors and inhibited by astrocytic factors [49], is known to contribute to inflammatory activity in ischemic stroke. Its suppression has been shown to reduce brain infarct progression after stroke [66], as observed here after DMF treatment.

Additionally, DMF treatment after MCAO resulted in lower levels of many other plasma cytokines including IL-12, IP-10, IL-1β, eotaxin, RANTES, IL-17, and MCP-1 at 72 h and/or 7 days post-infarct. Most of these cytokines/chemokines are either Th-1 type or pro-inflammatory factors that are normally involved in the activation and subsequent infiltration of peripheral immune cells into the brain and in the activation of resident brain microglial cells [4–7, 10–12, 15, 17, 18, 20–24, 26–28, 32, 49, 67]. Of particular interest is IL-17A, which is produced primarily by Th17 cells and contributes widely to inflammation and the severity of clinical symptoms in autoimmune diseases like MS and other inflammatory diseases [27, 68, 69]. IL-17A promotes the production of inflammatory cytokines and chemokines, leading to the recruitment of IL-17A receptor-expressing immune cells such as neutrophils and macrophages. After stroke and breakdown of the blood-brain barrier (BBB), there is a massive infiltration of these activated immune cells into the brain, causing secondary brain injury and functional loss. Since DMF/MMF dramatically reduced the levels of IL-17 in plasma from MCAO rats and in OGD mixed cultures, it suggests that IL-17 may play an important role in ischemic stroke, although Th17 cell infiltration and intervention of IL-17 pathway were not directly studied here.

Concomitant with the reduction in these detrimental plasma and brain immune cytokines by DMF, we observed significantly fewer neutrophils and T-lymphocytes infiltrated into the infarct region, further lowering the levels of harmful cytokines in the brain [8, 13–16, 19–21, 24, 25, 28, 29, 67, 70]. Moreover, we found that DMF decreased activation of brain microglia/macrophages after ischemic stroke, similar to recent reports of the drug’s effects in models of hemorrhagic stroke in vitro [47] and in vivo [40, 44]. Taken together, these findings indicate that DMF acts as a potent immune modulator, down-regulating the further activation of microglia and reducing the infiltration of harmful blood immune cells that could further contribute to brain damage at later times after stroke. These beneficial immunomodulatory effects of DMF, which cannot be adequately explained by the activation of Nrf-2 antioxidant pathway, have been largely underappreciated in stroke. Given the potential clinical utility of the drug, further investigation into the mechanisms involved in DMF immunomodulation is needed.

The unique cytokine profiles in different tissues (i.e., plasma, brain, and nerve/glia cell cultures) elicited by DMF administration could provide valuable information in this regard. For instance, DMF strongly suppresses the IL-12 pathway and down-regulates IFN-γ and IP-10 in plasma, while it suppresses IFN-γ and IL-17 mRNA level in the mixed cell cultures, indicating a dual role in regulating IL-12 and IL-17 pathways. These could be important for therapeutic target development in the future. Moreover, the differences in tissue-specific cytokine profiles after DMF treatment underscore the importance of linking systemic responses with local brain responses, given the access of circulating peripheral cytokines to the brain after stroke and breakdown of the BBB [54].

Thus, systemically administered DMF may reduce brain inflammation via its direct effects on the peripheral immune system. Conversely however, DMF may also work indirectly to lower brain inflammation. After DMF treatment, there are fewer dying neurons in the brain, less activation of local glia/microglia, and fewer immune cells attracted to the region and likely a reduction in inflammatory cytokines leaking across the damaged BBB to blood, resulting in lower systemic levels of immune cytokines. Thus, DMF may reduce brain inflammation both through direct and indirect mechanisms [45].

In our studies, we observed the early action of DMF after experimental stroke compared to previous reports showing a late effect in MS [71]. There may be several reasons for these differences. In stroke but not MS, the BBB is extensively compromised, allowing rapid efficient access of the brain parenchyma to the drug. Interestingly, DMF was recently shown to improve BBB function after experimental stroke [45]. Also, in MS but not in stroke, infiltrated and activated resident immune cells exist in situ in the brain prior to DMF treatment which could slow the drug’s efficacy.

In summary, DMF-induced changes in the immune landscape result in the protection of brain tissue after MCAO, consistent with the observed decrease in infarct volume. As might be expected, the rescue of “at-risk” brain neurons in DMF-treated MCAO rats is correlated with fewer functional deficits and a sharp reduction in mNSS scores early on with a more gradual improvement in behavior over the next several weeks. Possibly, these differences reflect the early actions of DMF on Nrf-2 induction, leading to the rescue of core cells and a smaller infarct in the acute phase. This is followed by later downstream effects on HO-1, the slow rescue of penumbra cells, and a more gradual recovery of behavioral function at later times after stroke which is correlated with a decrease in immune cytokines in the brain and plasma, suggesting an important role for not only local but also the systemic immune system in ameliorating poststroke damage.

## Conclusions

This work shows that DMF, which is already safely used in the clinic to reduce relapse rate and disease progression in MS patients [42, 43], strongly suppresses multiple pro-inflammatory cytokines in in vivo and in vitro stroke models. The improvement of behavioral outcomes is consistent with the observed decrease in infarct volume after MCAO, likely due to the activation of Nrf-2 pathway and the antioxidant effects of DMF.

We conclude that DMF is neuroprotective in experimental stroke because of its dual capacity as an immunomodulatory and antioxidant agent and thus may be useful as a novel therapeutic reagent to treat stroke in patients. As we explore ways to translate this work from bench to bedside, ongoing studies will examine the therapeutic window for DMF efficacy and how we might exploit its underlying mechanisms to further impede damaging oxidant (i.e., HO-1) and/or inflammatory signaling pathways (i.e., IL-12 and/or IL-17).

## Additional file

Additional file 1: Figure S1. MMF partially rescues forebrain neurons from OGD-induced cell death in culture. Cultures of E15 rat forebrain were treated with MMF (25 μM) beginning after OGD deprivation and throughout the remaining culture period. Cultures treated with MMF contained fewer dead/dying cells at 24 h than untreated controls as assessed by LIVE/DEAD assay (A-C). No significant difference was observed in assay of LDH released into the media by dead cells (D). **p* < 0.05, Student’s *t* test. (TIF 2483 kb)

## Abbreviations

DMF: Dimethyl fumarate
GM-CSF: Granulocyte-macrophage colony-stimulating factor
HO-1: Heme oxygenase-1
IFNγ: Interferon gamma
IP-10: Interferon gamma-induced protein 10
MCAO: Middle cerebral artery occlusion
MCP1: Monocyte chemoattractant protein 1
MIP2: Macrophage inflammatory protein 2
MMF: Monomethylfumarate
mNSS: Modified neurological severity score
MPO: Myeloperoxidase
MS: Multiple sclerosis
Nrf2: (Erythroid-derived 2)-like 2
OGD: Oxygen-glucose deprivation
TNF-α: Tumor necrosis factor-alpha
VEGF: Vascular endothelial growth factor
