# Supplementary figures and images for: Fumarate modulates the immune/inflammatory response and rescues nerve cells and neurological function after stroke in rats
Source: J Neuroinflammation. 2016 Oct 13;13:269. doi: 10.1186/s12974-016-0733-1 (PMC5062839; doi:10.1186/s12974-016-0733-1)

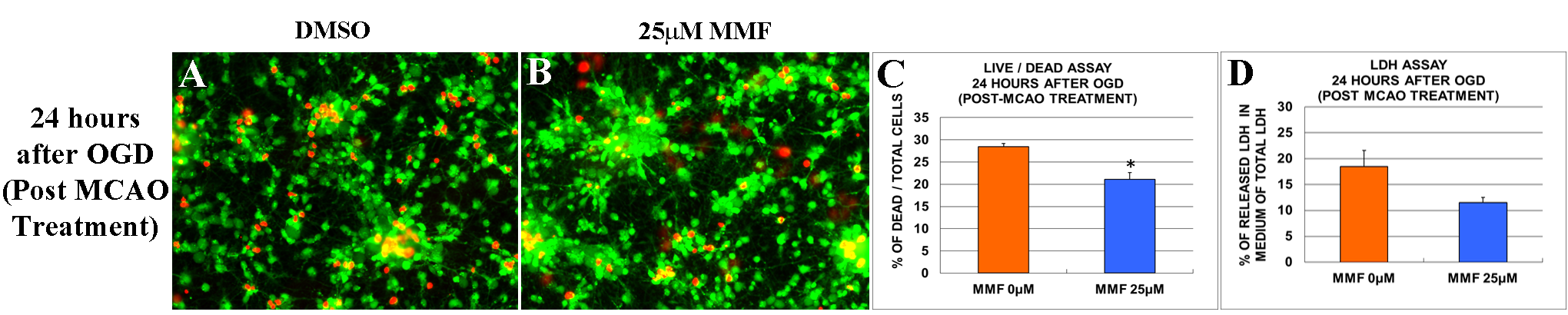

Supplement: Additional file 1: Figure S1. — MMF partially rescues forebrain neurons from OGD-induced cell death in culture. Cultures of E15 rat forebrain were treated with MMF (25 μM) beginning after OGD deprivation and throughout the remaining culture period. Cultures treated with MMF contained fewer dead/dying cells at 24 h than untreated controls as assessed by LIVE/DEAD assay (A-C). No significant difference was observed in assay of LDH released into the media by dead cells (D). *p < 0.05, Student’s t test. (TIF 2483 kb) [file 12974_2016_733_MOESM1_ESM.tif]
